# The effects of oral sodium bicarbonate supplementation on anthropometric measures in patients with chronic kidney disease: A systematic review and meta‐analysis of randomized clinical trials

**DOI:** 10.1002/fsn3.3627

**Published:** 2023-09-07

**Authors:** Fatemeh Navab, Mohammad Hossein Rouhani, Firouzeh Moeinzadeh, Cain C. T. Clark, Rahele Ziaei

**Affiliations:** ^1^ Student Research Committee, Nutrition and Food Security Research Center and Department of Community Nutrition, School of Nutrition and Food Science Isfahan University of Medical Sciences Isfahan Iran; ^2^ Nutrition and Food Security Research Center, Department of Community Nutrition, School of Nutrition and Food Science Isfahan University of Medical Sciences Isfahan Iran; ^3^ Isfahan Kidney Diseases Research Center Isfahan University of Medical Sciences Isfahan Iran; ^4^ Institute for Health and Wellbeing Coventry University Coventry UK

**Keywords:** anthropometric, chronic kidney disease, metabolic acidosis, muscle wasting, sodium bicarbonate

## Abstract

Metabolic acidosis (MA) may play a key role in the pathogenesis of protein‐energy wasting (PEW) in patients with chronic kidney disease (CKD). To present a comprehensive synthesis of the effect of oral sodium bicarbonate (SB) supplementation on anthropometric measures in patients with CKD, a systematic review was undertaken in PubMed/MEDLINE, Web of Science, Cochrane CENTRAL, and Google Scholar, of relevant articles published prior to September 2022. The summary statistics of effect size, nonstandardized weighted mean difference (WMD), and 95% confidence interval (CI) were used to compare the effects of SB supplementation on anthropometric parameters vs. control group. To detect probable sources of heterogeneity, a series of predefined subgroup analyses were conducted. In total, 17 studies with 21 treatment arms, including 2203 participants (1149 cases, 1054 controls), met our inclusion criteria and were included in the meta‐analysis. SB supplementation had no significant effect on body weight (BW), midarm muscle circumference (MAMC), or lean body mass (LBM) in patients with CKD. There was a significant increase in body mass index (BMI) (MD: 0.59 kg/m^2^, 95% CI: 0.25 to 0.93, *p* = 0.001) after SB supplementation in the overall analysis. In subgroup analysis, LBM was increased in studies that were ≥ 24‐week duration (MD: 1.81 kg, 95% CI: 0.81 to 2.81) and in participants with BMI lower than 27 kg/m^2^ (MD: 1.81 mg/L, 95% CI: 0.81 to 2.81). SB supplementation may yield increases in BMI in predialysis CKD patients. However, our findings did not support the beneficial effects of SB supplementation on other anthropometric outcomes. There is an evident need for long‐term high‐quality interventions to confirm these findings.

## Graphical abstract

Sodium bicarbonate supplementation may be efficacious in increasing BMI in predialysis CKD patients. However, our findings did not support the beneficial effects of sodium bicarbonate supplementation on other anthropometric outcomes.

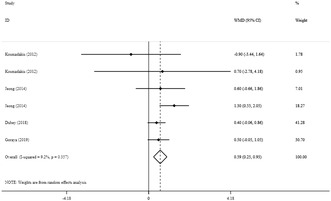

## 1 INTRODUCTION

Chronic kidney disease (CKD), defined as decreased kidney function of at least 3‐month duration, has become one of the leading causes of death around the world (Bradshaw et al., 2019; GBD 2017 Causes of Death Collaborators, 2018). CKD can lead to a variety of complications including cardiovascular diseases, renal osteodystrophy, anemia, and metabolic acidosis (MA) (Chen et al., 2019; Reiss et al., 2018). Increased mortality and morbidity of the disease are mainly attributable to these complications (Cheng et al., 2021).

About 15% of patients with CKD suffer from metabolic acidosis, defined as serum sodium bicarbonate level lower than 22 mmol/L (Raphael et al., 2014; Wesson et al., 2020). Metabolic acidosis occurs due to the impaired renal ability to regulate acid–base balance, as hydrogen ions cannot be excreted and bicarbonate cannot be produced to counteract metabolic acidification, following renal insufficiency (Yaqoob, 2010). In patients with CKD, MA may accelerate impairment of renal function and consequently play a key role (Menon et al., 2010; Raphael et al., 2011, 2013) in the pathogenesis of some CKD complications, including insulin resistance, cardiovascular disease, decreased protein synthesis, increased protein catabolism and branched‐chain amino acid oxidation, low leptin levels, and protein‐energy wasting (PEW) (Kalantar‐Zadeh et al., 2004; Kopple et al., 2005; Rajan & Mitch, 2008; Stenvinkel et al., 2002).

Based on findings from a recent meta‐analysis, 11%–54% of patients with CKD stages 3–5 suffer from PEW (Carrero et al., 2018). Protein‐energy wasting, along with cachexia and muscle wasting, is closely associated with CKD mortality and morbidity (Obi et al., 2015). Moreover, PEW in CKD may be related to uremic toxins, hypercatabolic status, inflammation, endocrine disorders, and MA (Carrero et al., 2013; Wang & Mitch, 2014). Metabolic acidosis, by inducing insulin resistance, may lead or contribute to lean body mass loss (Wang & Mitch, 2014).

It has long been established that conservative treatment of CKD should aim at correcting metabolic acidosis to prevent or reverse PEW and muscle wasting (Chiu et al., 2009; Chiu & Mehrotra, 2010). As a part of CKD management, oral bicarbonate‐based therapy has been used to neutralize MA for many years (Hu et al., 2019). Based on clinical studies, the effectiveness of sodium bicarbonate (SB) to correct acid–base disturbances in CKD patients is more pronounced than sodium citrate (Chen & Abramowitz, 2019; Raphael, 2016). According to the 2012 Kidney Disease Improving Global Outcomes guidelines, oral bicarbonate supplementation for CKD patients with serum bicarbonate concentration lower than 22 mEq/L is recommended (Improving Global Outcomes Lipid Guideline Development Work Group, 2013).

Despite the widespread use of SB in the treatment of metabolic acidosis in CKD (Goraya et al., 2013, 2014; Łoniewski & Wesson, 2014), it is not clearly defined whether managing metabolic acidosis may affect nutritional parameters. To summarize the evidence on this topic, we conducted a systematic review and meta‐analysis to evaluate the efficacy of oral sodium bicarbonate supplementation on anthropometric measures, including body weight (BW), body mass index (BMI), lean body mass (LBM), and midarm muscle circumference (MAMC) in predialysis CKD patients.

## 2 METHODS

### 2.1 Literature search and selection

We conducted a systematic review and meta‐analysis in accordance with the Preferred Reporting Items for Systematic Reviews and Meta‐Analysis (PRISMA) guidelines, and according to the PICOS criteria (Population: adults; Intervention: use of oral SB supplementation; Comparator: placebo/no treatment; Outcomes: BW, BMI, LBM, and MAMC; Study design: RCTs and the below predefined inclusion and exclusion criteria to identify potential eligible trials) to assess the impact of SB supplementation on anthropometric measures in patients with CKD. A systematic search of studies published between 2000 until September 24, 2022 was performed on PubMed/MEDLINE, Web of Science, Cochrane CENTRAL, and Google Scholar. Electronic searches were complemented by hand searches of the reference lists of eligible articles. Search terms were a combination of keywords relevant to sodium bicarbonate and study design to identify related publications. Further details about the search strategy are provided in Table S1. Search results were limited to human randomized controlled trials. Initial screening of RCTs included a review of titles and abstracts (first pass screening) followed by full‐text review (second pass screening) as necessary to determine eligibility for inclusion. Two reviewers (F.S.N and R.Z) independently screened titles and abstracts according to the PICOS criteria.

### 2.2 Inclusion and exclusion criteria

Relevant articles were included if they: (1) compared oral bicarbonate supplementation therapy with placebo, usual patient care or no study medication on predialysis CKD patients; (2) provided sufficient information including mean, standard deviation (SD), and the number of participants in each study of change in LBM, BW, MAMC, and BMI across study arms or reported sufficient information to estimate those values; (3) were randomized controlled trial; (4) be available as a full‐text publication; and (5) were published in any language. The exclusion criteria were as follows: (1) reported duplicate data (in these cases, the study with the largest sample size was included); (2) involved either dialysis or kidney transplantation patients; (3) evaluated the effects of intravenous SB supplementation; (4) were conducted in participants under the age of 18 years (children and adolescents) or in pregnant or lactating women; (5) unable to extract the corresponding information; (6) did not have placebo or untreated control groups; and (7) were reviews, letters, editorial articles, case reports, observational studies, books, conferences, or nonrandomized trials.

### 2.3 Quality assessment

Using Cochrane Collaboration's tool 1 (ROB1) (Higgins et al., 2011), the risk of bias in selected articles was assessed by two independent authors (R.Z and F.S.N), based on the following seven domains: (1) blinding of participants, personnel, and investigators, (2) blinding of outcome assessors, (3) random sequence generation, (4) allocation concealment, (5) incomplete outcome data, (6) selective outcome reporting, and (7) other sources of bias. Depending on the bias degree in each domain, it is categorized as either low, high, or unclear. The overall quality of each study was considered as ‘good’ if more than two items were low risk, ‘fair’ if only two items were low risk, and ‘weak’ if less than two items were low risk.

### 2.4 Data extraction

Related data were extracted by two independent investigators (R.Z and F.S.N). The following relevant data were extracted using a standard data extraction sheet: first author, year of publication, study location, health status of study participants, number of participants, study design, sex of participants, duration of study, form of SB and placebo administration, type of comparator, background diet, dosage of SB administration, and outcomes. When the data were reported at multiple measurements, only the outcomes at the end of the intervention were included in the analysis. In the case of multiple publications with duplicate and/or overlapped data for the same trial, the publication with most comprehensive and complete data was selected. Data were cross‐checked to minimize potential errors, and disagreements were resolved through discussion with the third author (S.M.H.R).

### 2.5 Statistical analysis

All statistical analyses were carried out using STATA version 17 (Stata Corp, College Station). Mean change of outcomes within intervention groups and placebo groups was calculated by subtracting the mean values at the end of follow‐up from those at the baseline. Comparison between SB groups and control groups was obtained by subtracting calculated differences within intervention groups from control groups. Calculation of the standard deviations (SDs) was conducted using the following formula: SD = square root ([SD pretreatment]2 + [SD posttreatment]2‐[2R × SD pretreatment × SD posttreatment]), using a correlation coefficient of the change‐value SDs that were available from the other studies based on the presented method in the Cochrane guidelines (Higgins & Green, 2011). To calculate standard deviation (SD) in trials reporting standard error of mean (SEM), the following formula was used: SD = SEM × square root of the number of participants. Mean differences and standard deviation in different types of anthropometric indices were computed for all trials. The summary statistics of effect size, nonstandardized weighted mean difference (WMD), and 95% confidence interval (CI) were used to compare the effects of SB supplementation on anthropometric parameters vs. control group. Heterogeneity was assessed by using Cochrane's Q and *I* ^*2*^ statistic values. An *I* ^*2*^ value <50% indicated nonsignificant heterogeneity, a value ≥50% was considered as moderate heterogeneity, and a value ≥75% was considered as substantial heterogeneity between studies. To detect probable sources of heterogeneity, a series of predefined subgroup analyses were conducted based on health status (CKD 1–2, CKD 3–5, CKD with diabetes, CKD 1–2 with hypertension, and CKD 3–5 with hypertension), age (<55 years old vs. ≥55), BMI (<27 kg/m^2^ vs. ≥27), dose of SB (<0/7 mEq/kg bw/day vs. ≥0/7 mEq/kg bw/day), and follow‐up duration (<44 weeks vs. ≥44 or < 24 weeks vs. ≥24 weeks). Influence analysis was conducted to test the potential impact of each trial on the pooled effect size. The potential for publication bias was tested using Egger's test, Begg's test, and by visual inspection of funnel plots. Also, we examined possible publication bias using significance funnel plots, Figure S1. *p* < 0.05 was used to demarcate statistical significance.

## 3 RESULTS

### 3.1 Literature search

**FIGURE 1:**
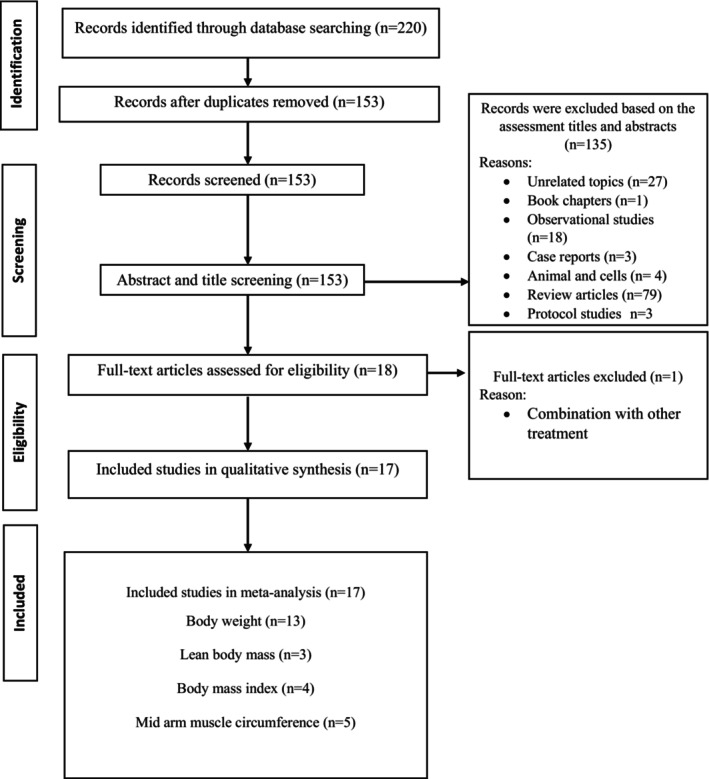
Flow chart of the process of the study selection.

We identified 220 publications through initial electronic searches. Of those, 67 were removed based on duplicates. In the next step, 153 trials remained for screening of the title and abstract, of which, 135 papers were excluded due to unrelated topics, book chapters, animal and cell studies, observational studies, case reports, review studies, and protocol studies. Next, out of 18 articles, after their full texts had been checked, one paper was excluded because of cotreatment. Ultimately, 17 studies met our inclusion criteria. Details of the included studies extracted for analysis are shown in Table 1. The study selection process is shown in Figure 1.

**TABLE 1:** Characteristics of eligible studies examining the effect of sodium bicarbonate supplementation on anthropometric measures in predialysis CKD patients.

Study (year) ref	Participant characteristics					Study characteristics										
	Population	Sample size	Gender (M/F)	Mean BMI (kg/m^2^)	Mean Age (years)	Baseline/End of treatment serum HCO3 (mmol/L)	Measured outcomes	Location	RCT design	Duration (weeks)	Blinding	Background Diet	Dose (mEq/kg bw/day)	Form of Administration	Comparator	Background Diet
BiCARB study group (2020) Witham et al. (2020)	CKD4,5	161	Both	28.6	74	20.35/ND	BW/MAMC	UK	P	96	TB	No trial‐specific dietary advice	Initiated at 0.5 mmol/kg/day (Up to 3 g/day)	Tablet	Placebo tablet	No trial‐specific dietary advice
Gaggl (2021) Gaggl et al. (2021)	CKD3,4 and chronic metabolic acidosis	47	Both	28.1	57	19/24	BW	Austria	P	8	UB	ND	0.750	ND	Standard care	ND
Melamed (2020) Melamed et al. (2020)	CKD3,4	149	Both	33.6	61	24/ND	BW	USA	P	96	TB	ND	0.4 mEq/IBW/day	Capsule	Placebo capsule	ND
Raphael (2020) Raphael, Greene, et al. (2020)	CKD with DM	74	Both	33	72	ND	BW	USA	P	24	DB	ND	0.5 meq/kg lean body wt/day	Pill	Placebo	ND
Di Iorio (2019) Di Iorio et al. (2019)	CKD3‐5 with metabolic acidosis	740	Both	27.9	67.8	21.5/24	BW	Italy	P	144	UB	Low pr, Low *p* Low Na	First year:1.13 (0.10); second year: 1.12 (0.11); third year: 1.09 (0.12)	ND	Standard care	Low pr, Low P Low Na
Kittiskulnam (2019) Kittiskulnam et al. (2020)	CKD3,4	42	Both	28	61.2	21/24–26	BW/LBM/MAMAC	Thailand	P	16	ND	Low pr, Low Na	ND	Tablets (Sodamint®)	Adjusted dosage	Low pr, Low Na
Bellasi (2016) Bellasi et al. (2016)	CKD with DM2	145	Both	ND	65.5	21.4/24.2	BW	Italy	P	48	UB	ND	0.7 ± 0.2 (0.5)	ND	Conventional therapy	ND
Goraya (2012) Goraya et al. (2012)	CKD1,2	133	Both	ND	50.45	ND	BW	USA	P	4	UB	Ad lib diets	0.5	Tablets	ND	Ad lib diets
Goraya (2014) Goraya et al. (2014)	CKD 3 with hypertensive nephropathy and metabolic acidosis	72	Both	ND	53.7	ND	BW	USA	P	144	UB	ND	0.3	Tablets	Usual Care	ND
Mathur (2006) Mathur et al. (2006)	Mild to moderate CKD	40	Both	ND	40.5	19.42/20.41	BW	India	P	12	SB	ND	1.2	Powder	Glucose	ND
Dubey (2018) Dubey et al. (2020)	CKD3,4	188	both	21.2	50.2	18.10/24—26	BW/LBM/BMI/MAMC	India	P	24	SB	A model diet	0.5	Generic tablets	Usual care	A model diet
Goraya (2019) Goraya et al. (2019)	CKD 3 with hypertensive nephropathy and metabolic acidosis	72	Both	28.25	53.75	22.97/ND	BMI	USA	P	240	UB	ND	0.3	Tablets	Usual care	ND
Raphael (2020) Raphael, Isakova, et al. (2020)	CKD3,4	194	Both	32.5	67	24/ND	BW	USA	P	28	DB	ND	Up to 96 kg:0.8 meq/kg lean body wt/day 78 kg:1 meq/kg lean body wt/day	Capsules	Cornstarch	ND
de Brito‐Ashurst (2009) de Brito‐Ashurst et al. (2009)	CKD4,5	129	Both	ND	54.77	19.8/≥23	MAMC	UK	P	96	DB	Increase calories in both groups	21.665 ± 9.523 meq/day	Tablets	Standard care	Increase calories in both groups
Jeong (2014) Jeong et al. (2014)	CKD4,5	73	Both	23.06	54.55	18.5/>22	BW/BMI/MAMC	Korea	P	48	UB	ND	0.58 ± 0.42	Tasna®	Standard care without alkali	ND
Alva (2020) Alva et al. (2020)	CKD4	58	Both	22.94	72.6	16.73/>23	LBM	India	P	First:24 S:36	ND	ND	21.427 meq/day	ND	Standard therapy	ND
Kosmadakis (2012) Kosmadakis et al. (2012)	CKD 4 or 5	32	Both	28.4	58	24.5/26.6	BMI	UK	P	24	UB	ND	ND	ND	Usual bicarbonate therapy	ND

Abbreviations: BMI, body mass index; BW, body weight; CKD, chronic kidney disease; DB, double blinded; DM, diabetes mellitus; IBW, ideal body weight; LBM, lean body mass; MAMC, midarm circumference; Meq, milliequivalent; ND, not determined; P, parallel; Pr, protein; RCT, randomized clinical trial; SB, single blinded; UB, unblinded; UK, United Kingdom; USA, United States of America; Wt, weight.

### 3.2 Characteristics of included studies

In total, 17 studies with 21 treatment arms, including 2203 participants (1149 cases, 1054 controls), met our inclusion criteria and were included in our meta‐analysis. All included studies were of parallel design. Among the 17 RCTs, two (11.7%) included participants with CKD stages 1 and 2, 11 studies (64.7%) included participants with CKD stages 3–5, two studies (11.7%) included participants with CKD and diabetes, and two studies (11.7%) included participants with CKD and hypertension. The mean age of participants in RCTs ranged from 49 to 67 years and had a baseline BMI ranging from 21.2 to 33.6 kg/m^2^. Furthermore, the intervention duration of the included studies ranged from 4 to 240 weeks. RCTs included for analysis were published between 2006 and 2021 and included the following countries of origin: six studies (35.2%) were conducted in Europe, six studies (35.2%) were conducted in North America, and five studies (29.4%) were conducted in Asia. The intervention dose of sodium bicarbonate varied from 0.4 to 1.2 mEq/kg bw/day, depending on the prescribed form of sodium bicarbonate.

### 3.3 Meta‐analysis results

#### 3.3.1 Body weight

**FIGURE 2:**
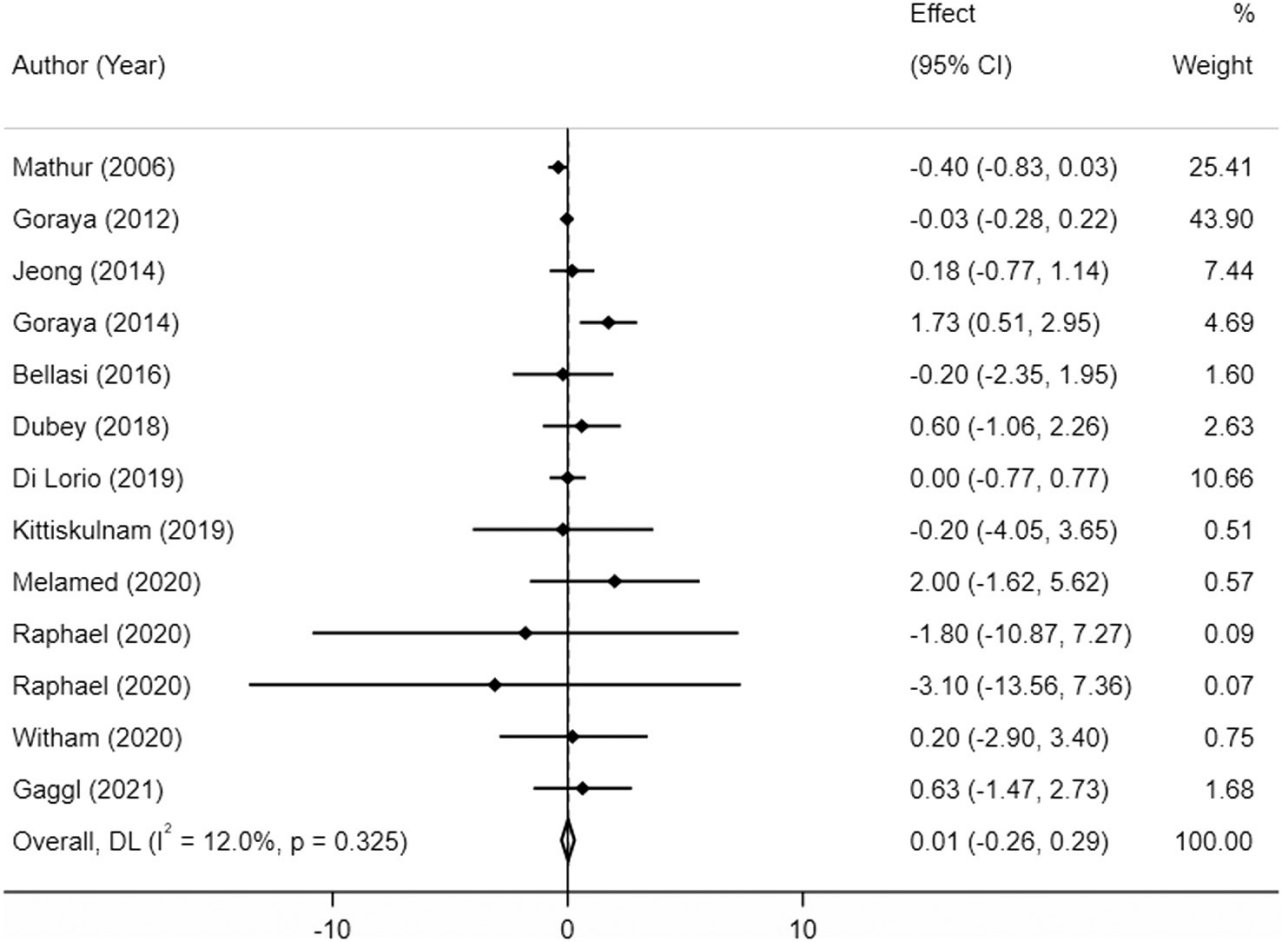
The effects of sodium bicarbonate supplementation on BW.

The effect of sodium bicarbonate supplementation on body weight (BW) was examined in 13 studies with 15 treatment arms, containing 1484 subjects (1041 cases, 443 control). The pooled analysis demonstrated that sodium bicarbonate supplementation yielded no significant effect on BW compared to placebo (MD: 0.01 kg, 95% CI: −0.26 to 0.29, *p* = 0.84 and 0.16 kg, 95% CI: −0.31 to 0.64, *p* = 0.52, overall and in studies with less than 12‐week duration, respectively) and between‐study heterogeneity was low (*I* ^2^ = 19.2%, *p* = 0.32; Table S2; Figure 2).

Sensitivity analysis suggested that no individual trial had a substantial impact on the overall pooled effect size. Begg's test (*p* = 0.661) and Egger's test (*p* = 0.339) suggested no evidence of publication bias.

#### 3.3.2 Body mass index

**FIGURE 3:**
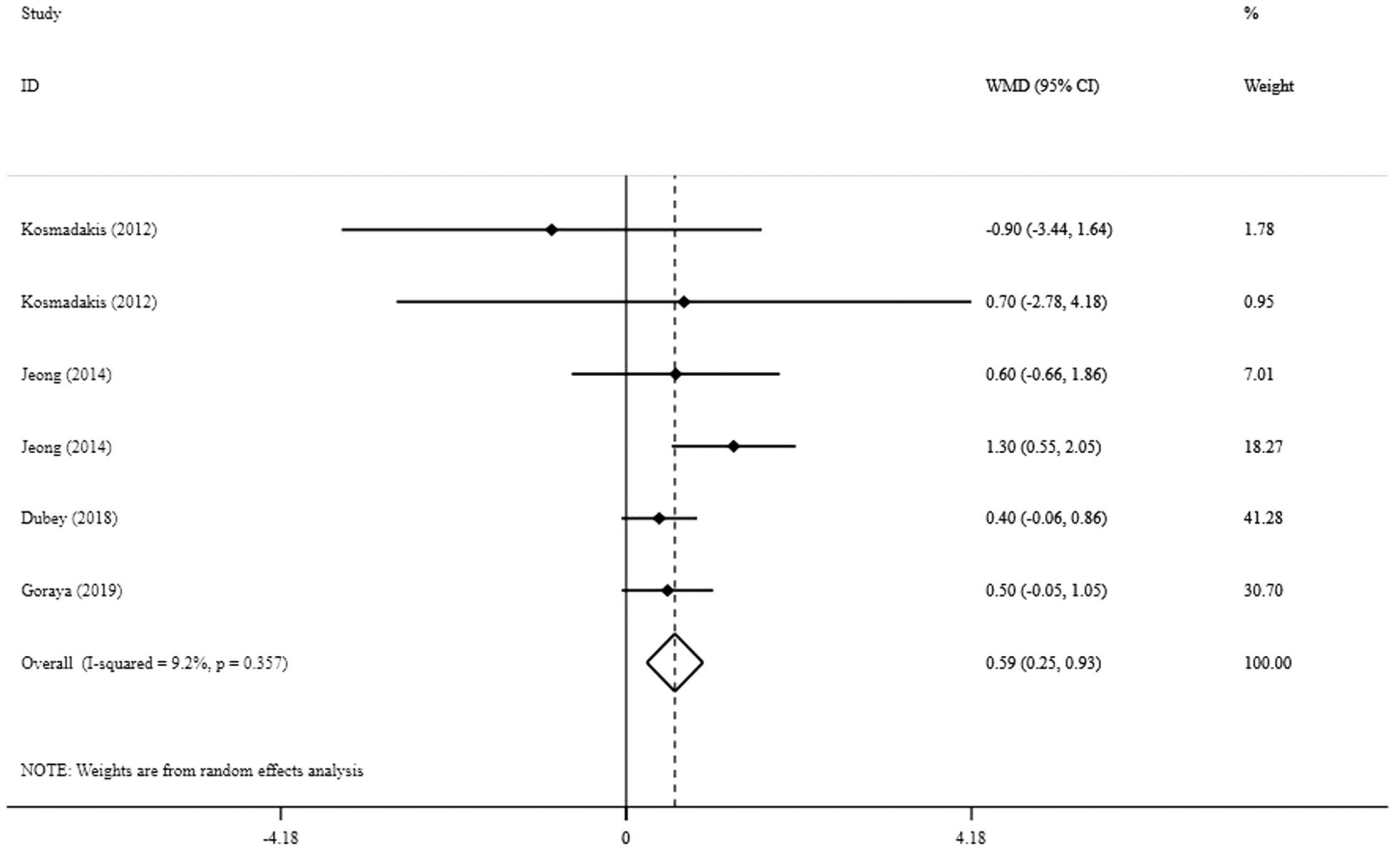
The effects of sodium bicarbonate supplementation on BMI.

Overall, four eligible studies with six treatment arms, including a total of 365 participants (183 cases, 182 control), examined the effect of sodium bicarbonate supplementation on BMI. Body mass index was significantly increased after sodium bicarbonate supplementation compared to the control group (MD: 0.59 kg/m^2^, 95% CI: 0.25 to 0.93, *p* = 0.001), with low between‐study heterogeneity (*I* ^2^ = 9.2%, *p* = 0.35) (Figure 3).

Additional analysis showed a greater increase in BMI in trials with a longer duration (≥44 weeks) (MD: 0.76 kg/m^2^, 95% CI: 0.34 to 1.19) compared to the short duration (<44 weeks), and when conducted in people with BMI lower than 27 kg/m^2^ (MD: 0.64 mg/dL, 95% CI: 0.27 to 1.01) compared to those with BMI ≥ 27 kg/m^2^ (Table S2).

Sensitivity analysis suggested that no individual trial had a substantial impact on the overall pooled effect size. Furthermore, there was no indication of publication bias using the Egger's test (*p* = 0.5) or Begg's test (*p* = 0.3).

### 3.4 Midarm muscle circumference

**FIGURE 4:**
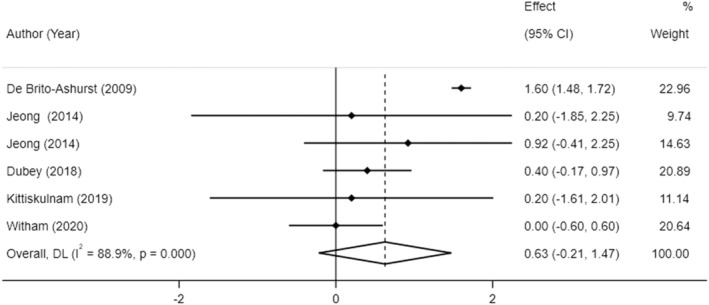
The effects of sodium bicarbonate supplementation on MAMC.

The effect of sodium bicarbonate supplementation on MAMC was investigated in five RCTs, including six treatment arms, with 593 participants (294 cases, 299 control). Pooled results indicated that sodium bicarbonate supplementation had no significant effect on MAMC compared to placebo (MD: 0.63 cm, −0.21 to 1.47, *p* = 0.14; Figure 4). Significant heterogeneity was observed across studies (*I* ^2^ = 81.2%, *p* < 0.001). The study duration and baseline BMI were possible sources of heterogeneity (Table S2).

Sensitivity analysis suggested that overall estimates for MAMC were not affected by the removal of any individual study. No statistically significant publication bias was evident (*p* = 0.8, Begg's test and *p* = 0.1, Egger's test).

### 3.5 Lean body mass

**FIGURE 5:**
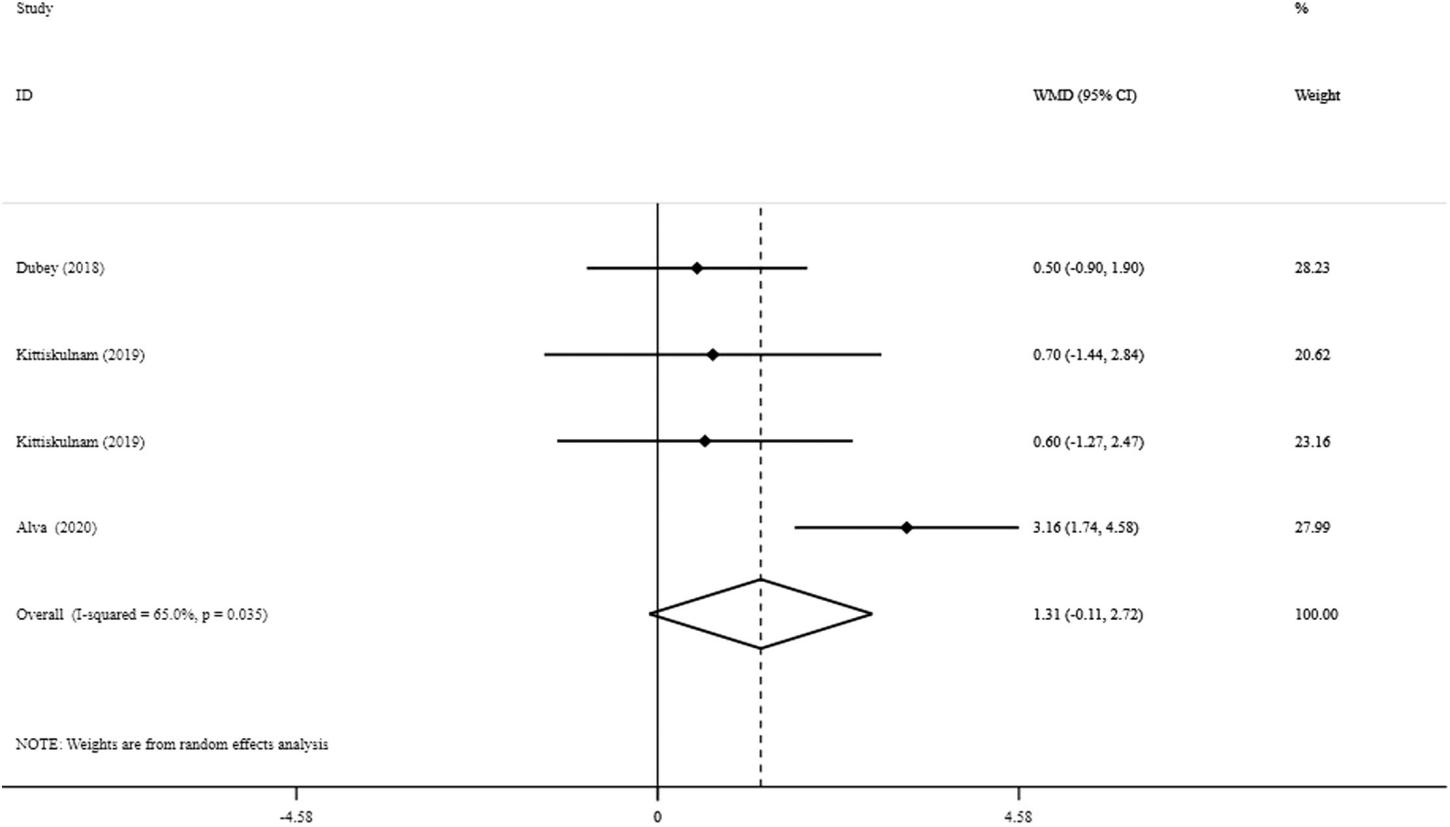
The effects of sodium bicarbonate supplementation on LBM.

Using data from three studies, with four treatment arms, containing 309 participants (166 cases and 143 controls), meta‐analysis suggested that sodium bicarbonate supplementation had no significant effect on LBM (MD: 1.31 kg, 95% CI: −0.11 to 2.72, *p* = 0.07) and that between‐study heterogeneity was significant (*I* ^2^ = 65%, *p* = 0.03) Figure 5.

Subgroup analysis suggested that the effect size was greater in studies that had a ≥ 24‐week duration (MD: 1.81 kg, 95% CI: 0.81 to 2.81) and in participants with BMI lower than 27 kg/m^2^ (MD: 1.81 mg/L, 95% CI: 0.81 to 2.81) (Table S2).

Sensitivity analyses revealed that the exclusion of any single study from the analysis did not alter the overall effect. Begg's test (*p* = 0.73) and Egger's test (*p* = 0.59) suggested no evidence of publication bias.

### 3.6 Risk‐of‐bias assessment

Based on the Cochrane risk‐of‐bias tool 1 (ROB 1), 12 studies (70.5%) were rated as having good overall quality and five studies (29.4%) had fair overall quality. Table S3 presents the quality of the studies.

### 3.7 Publication bias by significance funnel plot

Examining significance funnel plots (Figure S1), the summary estimates were robust to publication bias for BMI and BW, suggested by the close distance between the pooled estimates across effect sizes (the black diamond) and within only the nonaffirmative effect sizes (the gray diamond). In contrast, some evidence of publication bias was observed for LBM and MAMC.

## 4 DISCUSSION

The findings from the present systematic review and meta‐analysis indicate that oral sodium bicarbonate supplementation might improve BMI in predialysis CKD patients, especially in trials with longer duration and in participants with a starting BMI lower than 27 kg/m^2^. We found no significant effect of sodium bicarbonate supplementation on BW, MAMC, and LBM. However, in subgroup analysis, LBM was increased in studies with ≥24‐week duration and in participants with BMI lower than 27 kg/m^2^. To our knowledge, this is the first systematic review and meta‐analysis studying the effects of sodium bicarbonate supplementation on anthropometric measures and muscle mass specifically in patients with CKD.

Metabolic acidosis is a common complication of CKD, which can lead to increased inflammation, protein malnutrition, sarcopenia, PEW, acceleration of progression of CKD, and finally CKD morbidity and mortality. Metabolic acidosis is among the key mechanisms which have been proposed to explain adverse nutritional changes in these patients. Correction of MA with base administration was associated with improved kidney function and bone disease, increased insulin sensitivity, and decreased muscle wasting (Cheng et al., 2021; Kraut & Madias, 2017). In a systematic review and meta‐analysis on the effects of oral sodium bicarbonate on renal function and cardiovascular risk in patients with CKD, treatment of MA with sodium bicarbonate was associated with reduced decline rate of kidney function and improved endothelial function (Cheng et al., 2021).

No significant improvement of LBM and MAMC following SB supplementation was found in the present study, a finding which is consistent with the meta‐analysis by Cheng et al. (Cheng et al., 2021). We found that LBM may improve in long‐term studies and when baseline BMI was <27 kg/m^2^. However, due to limited number of studies evaluating LBM and their heterogeneity in LBM measurement methods: bioimpedance analysis (Kittiskulnam et al., 2020), DXA scan (Dubey et al., 2020), and equation based on corrected arm muscle area (Alva et al., 2020), our findings should be interpreted with caution in this regard. Loss of cellular protein and decreased protein content in the body, leading to muscle atrophy, increases morbidity and mortality in patients with CKD (Wang & Mitch, 2014). Thus, therapeutic approaches which inhibit or block protein loss can prevent CKD‐induced muscle wasting and improve CKD prognosis. CKD‐induced protein loss cannot be reversed by simply increasing dietary protein and energy, suggesting that catabolic pathways might be involved (Cano et al., 2007). In this regard, ubiquitin–proteasome system (UPS), caspase‐3, lysosomes, and myostatin are among suggested catabolic pathways (Wang et al., 2022; Wang & Mitch, 2014). Metabolic acidosis, insulin resistance, hormonal changes, inflammatory processes, and decreased dietary intake may initiate the activation of these pathways. Several mechanisms have been proposed to explain the beneficial effects of targeting MA on protein status of the body and muscle mass. Promoting muscle mitochondrial function, decreasing insulin resistance, and suppressing protein catabolic pathways are among such mechanisms (Chalupsky et al., 2021).

No significant improvement of BW was observed in the present study, a finding which is consistent with a similar meta‐analysis investigating the effects of SB on cardiovascular risk (Cheng et al., 2021). In our study, we observed that BMI may increase in predialysis CKD patients following SB supplementation; however, the discrepancy observed between the findings for BW and BMI (no significant increase in BW vs. significant improvement in BMI following alkali administration), potential sodium‐mediated fluid retention following long‐term SB supplementation, and limited number of studies included for BMI should be taken into consideration when interpreting the results for BMI (Abramowitz et al., 2013). Although obesity is considered an important risk factor for new‐onset kidney disease, in patients with CKD the evidence is challenging. In this regard, reduced risk of CKD progression and mortality, when BMI is in the overweight range, has been reported in several studies (Davis et al., 2016; Ladhani et al., 2017; Lu et al., 2014). In a large cohort study by Liu et al., investigating the association of BMI with CKD outcomes in 453,946 nondialysis‐dependent CKD patients, with an estimated glomerular filtration rate (eGFR) below 60 mL/min per 1.73 m^2^, a U‐shaped association between BMI and CKD progression and mortality was found, and overweight and mild obesity were associated with the best outcomes. This paradoxical benefit of obesity has also been reported in other disease conditions, such as chronic heart failure, diabetes, and chronic obstructive pulmonary disease (Curtis et al., 2005; Tseng, 2013). Although BMI is unable to discriminate body composition, higher BMI may reflect higher muscle mass and increased adaptation to PEW (Davis et al., 2016).

The present comprehensive systematic review and meta‐analysis is the first to explore the effects of oral sodium bicarbonate supplementation on anthropometric measures in predialysis CKD patients. We performed a comprehensive assessment of study quality and biases to minimize the presence of confounding variables and many of the studies included in this analysis were considered to be of high quality based on Cochrane Risk‐of‐Bias Tool. However, several potential limitations of this study should be noted. First, due to the limited number of studies included for BMI and LBM, our findings should be interpreted with caution for these outcomes. Second, although we performed several prespecified subgroup analyses to detect sources of heterogeneity, residual heterogeneity might persist within some subgroups. Third, since few studies have reported the data for fat mass, we were unable to include fat mass as an important anthropometric outcome in this study.

## 5 CONCLUSION AND FUTURE RESEARCH

Sodium bicarbonate supplementation may be efficacious in increasing BMI in predialysis CKD patients. However, our findings did not support the beneficial effects of SB supplementation on other anthropometric outcomes including BW, MAMC, and LBM. In subgroup analysis, LBM was improved in long‐term studies and in patients with BMI less than 27 kg/m^2^. There is an evident need for future long‐term high‐quality interventions to examine the effects of sodium bicarbonate on protein status and various anthropometric measures, including muscle mass and visceral and overall fat mass, as primary outcomes.

## AUTHOR CONTRIBUTIONS

**Fatemeh Navab:** Data curation (equal); methodology (equal); software (equal); writing – original draft (equal). **Mohammad Hossein Rouhani:** Conceptualization (lead); data curation (equal); methodology (equal); supervision (equal); writing – review and editing (lead). **Firouzeh Moeinzadeh:** Writing – review and editing (equal). **Cain C. T. Clark:** Writing – review and editing (supporting). **Rahele Ziaei:** Data curation (equal); formal analysis (lead); methodology (lead); supervision (lead); writing – original draft (equal).

## FUNDING INFORMATION

The present study was supported by a grant from Vice Chancellor for Research, Isfahan University of Medical Sciences.

## CONFLICT OF INTEREST STATEMENT

None.

## Supporting information


Appendix S1

## Data Availability

None.
