# Supplementary material for: The effects of oral sodium bicarbonate supplementation on anthropometric measures in patients with chronic kidney disease: A systematic review and meta‐analysis of randomized clinical trials
Source: Food Sci Nutr. 2023 Sep 7;11(11):6749–60. doi: 10.1002/fsn3.3627 (PMC10630834; doi:10.1002/fsn3.3627)
Supplement: Supplementary file 1 — Appendix S1 [file FSN3-11-6749-s001.docx]

**The effects of oral Sodium bicarbonate supplementation on anthropometric measures in patients with chronic kidney disease: A systematic review and meta-analysis of randomized clinical trials**

Fatemeh Navab, Mohammad Hossein Rouhani, Firouzeh Moeinzadeh, Cain C. T. Clark, Rahele Ziaei

**Running Title:** Sodium bicarbonate and anthropometric measures in chronic kidney disease

| **Supplementary Table 1.** Search strategies including the key terms and the queries for each database | |
| --- | --- |
| **Database** | **Key terms and the queries** |
| **PubMed** | (((((((((("chronic renal insufficiency"[Title/Abstract]) OR ("chronic kidney insufficiency"[Title/Abstract])) OR ("chronic kidney disease"[Title/Abstract])) OR ("chronic kidney diseases"[Title/Abstract])) OR ("chronic renal disease"[Title/Abstract])) OR ("chronic renal diseases"[Title/Abstract])) OR ("Renal Insufficiency, Chronic"[Title/Abstract])) OR ("Kidney Insufficiencies, Chronic"[Title/Abstract])) OR ("Disease, Chronic Kidney"[Title/Abstract])) AND ((("sodium bicarbonate"[Title/Abstract]) OR ("Bicarbonate, Sodium"[Title/Abstract])) OR ("Sodium Hydrogen Carbonate"[Title/Abstract]))) AND (((((((((("randomized controlled trial"[Title/Abstract]) OR (randomised[Title/Abstract])) OR (randomized[Title/Abstract])) OR (placebo[Title/Abstract])) OR ("intervention studies"[Title/Abstract])) OR (intervention[Title/Abstract])) OR ("controlled trial"[Title/Abstract])) OR (random[Title/Abstract])) OR (randomly[Title/Abstract])) OR (assignment[Title/Abstract])) |
| **Web of Science (ISI)** | TS=("sodium bicarbonate" OR "Bicarbonate, Sodium" OR "Sodium Hydrogen Carbonate" OR "Hydrogen Carbonate, Sodium" OR "Baking Soda" OR "Soda, Baking") AND TS=( "randomized controlled trial" OR "randomized" OR "placebo") AND TS=( "Renal Insufficiency, Chronic" OR "Chronic Renal Insufficiencies" OR "Renal Insufficiencies, Chronic" OR "Chronic Renal Insufficiency" OR "Kidney Insufficiency, Chronic" OR "Chronic Kidney Insufficiency" OR "Chronic Kidney Insufficiencies" OR "Kidney Insufficiencies, Chronic" OR "Chronic Kidney Diseases" OR "Chronic Kidney Disease" OR "Disease, Chronic Kidney" OR "Diseases, Chronic Kidney" OR "Kidney Disease, Chronic" OR "Kidney Diseases, Chronic" OR "Chronic Renal Diseases" OR "Chronic Renal Disease" OR "Disease, Chronic Renal" OR " Diseases, Chronic Renal" OR "Renal Disease, Chronic" OR "Renal Diseases, Chronic") |
| **Cochrane**  **CENTRAL** | sodium bicarbonate OR Bicarbonate, Sodium OR Sodium Hydrogen Carbonate OR Hydrogen Carbonate, Sodium OR Baking Soda OR Soda, Baking in Title Abstract Keyword AND Renal Insufficiency, Chronic OR Chronic Renal Insufficiencies OR Renal Insufficiencies, Chronic OR Chronic Renal Insufficiency OR Kidney Insufficiency, Chronic OR Chronic Kidney Insufficiency OR Chronic Kidney Insufficiencies OR Kidney Insufficiencies, Chronic OR Chronic Kidney Diseases OR Chronic Kidney Disease OR Disease, Chronic Kidney OR Diseases, Chronic Kidney OR Kidney Disease, Chronic OR Kidney Diseases, Chronic OR Chronic Renal Diseases OR Chronic Renal Disease OR Disease, Chronic Renal OR Diseases, Chronic Renal OR Renal Disease, Chronic OR Renal Diseases, Chronic in Title Abstract Keyword - (Word variations have been searched) |

**Supplementary Table 2.** Result of subgroup analysis of included studies in meta-analysis

| **Sub-grouped by** | **No. of trials** | **Effect size^1^** | **95% CI, P value** | **I^2^ (%)** | **P for heterogeneity** | | | **P for between**  **subgroup heterogeneity** |
| --- | --- | --- | --- | --- | --- | --- | --- | --- |
| **BW (All trials)** | **13** | **0.01** | **(-0.26, 0.29), 0.84** | **12** | **0.325** |  | | |
| Duration |  |  |  |  |  | | | 0.11 |
| < 40 weeks | 7 | -0.11 | (-0.32, 0.11), 0.32 | 0 | 0.71 | | |  |
| ≥ 40 weeks  ≥ 12 weeks | 6  11 | 0.47  0.16 | (-0.21, 1.14), 0.13  (-0.31, 0.64), 0.52 | 26.7  24.4 | 0.23  0.211 | | |  |
| Dose |  |  |  |  |  | | | 0.06 |
| < 0.7 mEq/kg bw/day | 7 | 0.44 | (-0.21, 1.1), 0.18 | 38.5 | 0.13 | | |  |
| ≥ 0.7 mEq/kg bw/day | 4 | -0.27 | (-0.64, 0.09), 0.14 | 0 | 0.64 | | |  |
| Baseline BMI |  |  |  |  |  | | | 0.75 |
| <27 | 2 | 0.29 | (-0.54, 1.11), 0.49 | 0 | 0.67 | | |  |
| ≥27 | 7 | 0.12 | (-0.56, 0.8), 0.73 | 0 | 0.92 | | |  |
| Age |  |  |  |  |  | | | 0.89 |
| < 55 y | 5 | 0.15 | (-0.36, 0.65), 0.56 | 65.2 | 0.02 | | |  |
| ≥ 55 y | 8 | 0.09 | (-0.56, 0.74), 0.79 | 0 | 0.96 | | |  |
| Study Population | | | | | | | | 0.28 |
| CKD 1, 2 | 1 | -0.4 | (-0.83, 0.03), 0.07 | - | - | | |  |
| CKD 3-5 | 9 | 0.19 | (-0.34, 0.71), 0.48 | 0 | 0.94 | | |  |
| CKD with DM | 1 | -0.2 | (-2.35, 1.95), 0.85 | - | - | | |  |
| CKD 1, 2 with HTN | 2 | 0.74 | (-0.97, 2.46), 0.76 | 86.9 | 0.006 | | |  |
| **LBM (All trials)** | **4** | **1.31** | **(-0.11, 2.72), 0.07** | **65** | **0.03** |  | | |
| Duration |  |  |  |  |  | | | 0.18 |
| < 24 weeks | 2 | 0.64 | (-0.77, 2.05), 0.37 | 0 | 0.94 | | |  |
| ≥ 24 weeks | 2 | 1.81 | (0.81, 2.81), <0.001 | 85.3 | 0.009 | | |  |
| Baseline BMI |  |  |  |  |  | | | 0.18 |
| <27 | 2 | 1.81 | (0.81, 2.81), <0.001 | 85.3 | 0.009 | | |  |
| ≥27 | 2 | 0.64 | (-0.77, 2.05), 0.37 | 0 | 0.94 | | |  |
| **BMI (All trials)** | **6** | **0.59** | **(0.25, 0.93), 0.001** | **9.2** | **0.35** | |  | |
| Duration |  |  |  |  |  | | | 0.2 |
| < 44 weeks | 3 | 0.36 | (-0.08,0.81), 0.11 | 0 | 0.6 | | |  |
| ≥ 44 weeks | 3 | 0.76 | (0.34, 1.19), <0.001 | 30.8 | 0.23 | | |  |
| Baseline BMI |  |  |  |  |  | | | 0.55 |
| <27 | 3 | 0.64 | (0.27, 1.01), 0.001 | 50.2 | 0.13 | | |  |
| ≥27 | 3 | 0.44 | (-0.09, 0.98), 0.1 | 0 | 0.56 | | |  |
|  |  |  |  |  |  | | |  |
| **MAMC (All trials)** | **6** | **0.63** | **(-0.21, 1.47), 0.143** | **88.9** | **<0.001** | |  | |
| Duration |  |  |  |  |  | | | 0.551 |
| < 44 weeks | 2 | 0.38 | (-0.16,0.92), 0.165 | 0 | 0.83 | | |  |
| ≥ 44 weeks | 4 | 0.76 | (-0.35, 1.86), 0.18 | 89.5 | <0.001 | | |  |
| Baseline BMI |  |  |  |  |  | | | 0.25 |
| <27 | 3 | 0.46 | (-0.04, 0.97), 0.072 | 0 | 0.75 | | |  |
| ≥27 | 2 | 0.02 | (-0.55, 0.59), 0.94 | 0 | 0.83 | | |  |
|  |  |  |  |  |  | | |  |
|  |  |  |  |  |  | | |  |

^1^Calculated by Random-effects model.

**^2^** BW, Body Weight; BMI, Body Mass Index; CKD, Chronic Kidney Disease; LBM, Lean Body Mass; MAMC, Mid-Arm Muscle Circumference

| **Supplementary Table 3.** Risk of bias assessment in randomized controlled trials | | | | | | | | |
| --- | --- | --- | --- | --- | --- | --- | --- | --- |
| Author (year) | Random Sequence generation | Allocation  concealment | Blinding of  participants and  personnel | Blinding of outcome  assessment | Incomplete  outcome  data | Selective outcome  reporting | Other  sources of  bias | Overall  Quality |
| Gaggl (2021) (1) | L | L | H | U | L | L | U | good |
| Melamed (2020) (2) | L | U | L | L | L | L | U | good |
| Raphael (2020) (3) | L | U | L | H | L | H | L | good |
| Di Iorio (2019) (4) | U | L | H | L | L | L | U | good |
| Kittiskulnam (2019) (5) | L | L | U | L | L | L | U | good |
| Bellasi (2016) (6) | U | L | U | U | L | L | U | good |
| Goraya (2012) (7) | U | U | H | U | U | L | L | fair |
| Goraya (2014) (8) | U | U | H | U | L | L | L | good |
| Mathur (2006) (9) | U | U | L | H | U | L | U | fair |
| Dubey (2018) (10) | L | L | U | L | L | L | U | good |
| Raphael (2020) (11) | L | U | L | H | L | U | U | good |
| de Brito-Ashurst (2009) (12) | U | L | L | L | L | L | L | good |
| Jeong (2014) (13) | U | U | U | U | L | L | U | fair |
| Alva (2020 ) (14) | L | U | U | U | U | L | U | fair |
| Goraya (2019) (15) | U | U | H | U | L | L | L | good |
| Kosmadakis (2012) (16) | U | U | H | U | L | U | L | Fair |
| BiCARB study group (2020) (17) | L | L | L | L | L | H | U | Good |

^1^ H, high risk of bias; L, low risk of bias; U, unclear risk of bias.

**Supplementary Figure 1.** Significance funnel plots for study outcomes


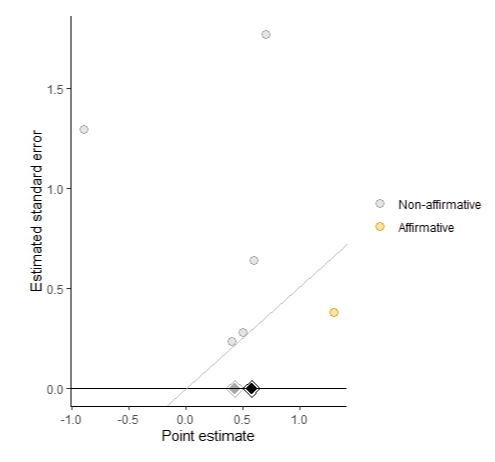

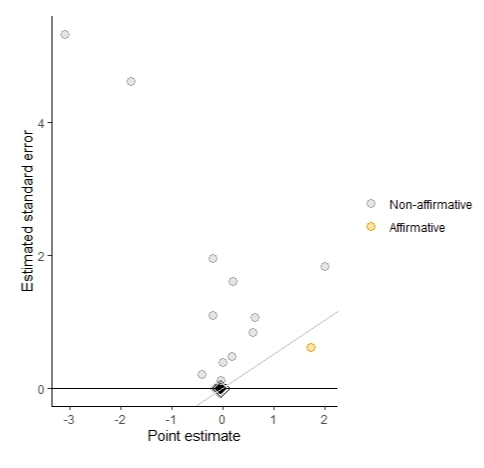


**BMI**

**BW**


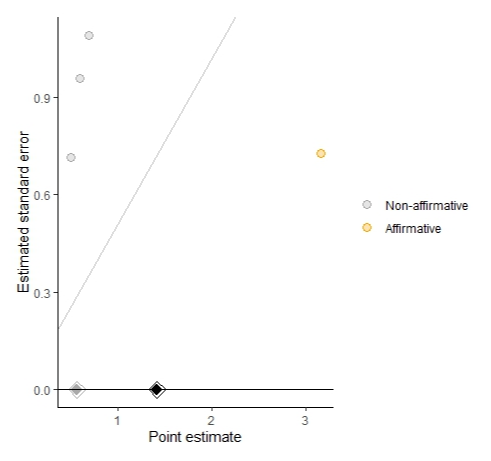

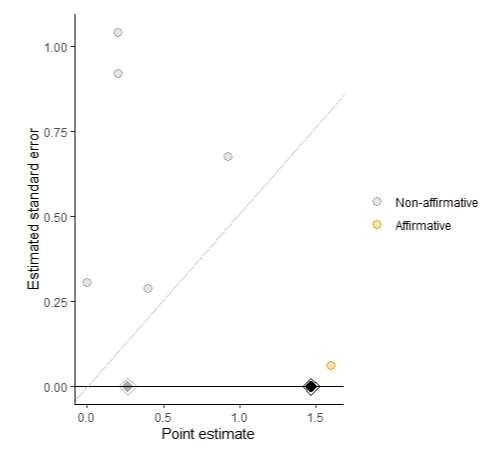


**MAMC**

**LBM**

^*^Point estimates are Fisher’s *z*-scale. Estimates lying on the diagonal line had *p*=0.05. Estimates were distinguished by whether they were ‘affirmative’ (i.e., *p*-value less than 0.05) or ‘non-affirmative’. Grey diamonds are robust clustered estimates among non-affirmative effect sizes only, and black diamonds are robust clustered estimates across all effect sizes.

**Supplementary References**

1. Gaggl M, Repitz A, Riesenhuber S, Aigner C, Sliber C, Fraunschiel M, et al. Effect of Oral Sodium Bicarbonate Treatment on 24-Hour Ambulatory Blood Pressure Measurements in Patients With Chronic Kidney Disease and Metabolic Acidosis. Front Med (Lausanne). 2021;8:711034.

2. Melamed ML, Horwitz EJ, Dobre MA, Abramowitz MK, Zhang L, Lo Y, et al. Effects of Sodium Bicarbonate in CKD Stages 3 and 4: A Randomized, Placebo-Controlled, Multicenter Clinical Trial. American journal of kidney diseases : the official journal of the National Kidney Foundation. 2020;75(2):225-34.

3. Raphael KL, Greene T, Wei G, Bullshoe T, Tuttle K, Cheung AK, et al. Sodium Bicarbonate Supplementation and Urinary TGF-β1 in Nonacidotic Diabetic Kidney Disease: A Randomized, Controlled Trial. Clinical journal of the American Society of Nephrology : CJASN. 2020;15(2):200-8.

4. Di Iorio BR, Bellasi A, Raphael KL, Santoro D, Aucella F, Garofano L, et al. Treatment of metabolic acidosis with sodium bicarbonate delays progression of chronic kidney disease: the UBI Study. Journal of nephrology. 2019;32(6):989-1001.

5. Kittiskulnam P, Srijaruneruang S, Chulakadabba A, Thokanit NS, Praditpornsilpa K, Tungsanga K, et al. Impact of Serum Bicarbonate Levels on Muscle Mass and Kidney Function in Pre-Dialysis Chronic Kidney Disease Patients. Am J Nephrol. 2020;51(1):24-34.

6. Bellasi A, Di Micco L, Santoro D, Marzocco S, De Simone E, Cozzolino M, et al. Correction of metabolic acidosis improves insulin resistance in chronic kidney disease. BMC nephrology. 2016;17(1):158.

7. Goraya N, Simoni J, Jo C, Wesson DE. Dietary acid reduction with fruits and vegetables or bicarbonate attenuates kidney injury in patients with a moderately reduced glomerular filtration rate due to hypertensive nephropathy. Kidney Int. 2012;81(1):86-93.

8. Goraya N, Simoni J, Jo CH, Wesson DE. Treatment of metabolic acidosis in patients with stage 3 chronic kidney disease with fruits and vegetables or oral bicarbonate reduces urine angiotensinogen and preserves glomerular filtration rate. Kidney Int. 2014;86(5):1031-8.

9. Mathur RP, Dash SC, Gupta N, Prakash S, Saxena S, Bhowmik D. Effects of correction of metabolic acidosis on blood urea and bone metabolism in patients with mild to moderate chronic kidney disease: a prospective randomized single blind controlled trial. Ren Fail. 2006;28(1):1-5.

10. Dubey AK, Sahoo J, Vairappan B, Haridasan S, Parameswaran S, Priyamvada PS. Correction of metabolic acidosis improves muscle mass and renal function in chronic kidney disease stages 3 and 4: a randomized controlled trial. Nephrol Dial Transplant. 2020;35(1):121-9.

11. Raphael KL, Isakova T, Ix JH, Raj DS, Wolf M, Fried LF, et al. A Randomized Trial Comparing the Safety, Adherence, and Pharmacodynamics Profiles of Two Doses of Sodium Bicarbonate in CKD: the BASE Pilot Trial. Journal of the American Society of Nephrology : JASN. 2020;31(1):161-74.

12. de Brito-Ashurst I, Varagunam M, Raftery MJ, Yaqoob MM. Bicarbonate supplementation slows progression of CKD and improves nutritional status. Journal of the American Society of Nephrology : JASN. 2009;20(9):2075-84.

13. Jeong J, Kwon SK, Kim HY. Effect of bicarbonate supplementation on renal function and nutritional indices in predialysis advanced chronic kidney disease. Electrolyte Blood Press. 2014;12(2):80-7.

14. Alva S, Divyashree M, Kamath J, Prakash PS, Prakash KS. A Study on Effect of Bicarbonate Supplementation on the Progression of Chronic Kidney Disease. Indian J Nephrol. 2020;30(2):91-7.

15. Goraya N, Munoz-Maldonado Y, Simoni J, Wesson DE. Fruit and Vegetable Treatment of Chronic Kidney Disease-Related Metabolic Acidosis Reduces Cardiovascular Risk Better than Sodium Bicarbonate. Am J Nephrol. 2019;49(6):438-48.

16. Kosmadakis GC, John SG, Clapp EL, Viana JL, Smith AC, Bishop NC, et al. Benefits of regular walking exercise in advanced pre-dialysis chronic kidney disease. Nephrol Dial Transplant. 2012;27(3):997-1004.

17. Clinical and cost-effectiveness of oral sodium bicarbonate therapy for older patients with chronic kidney disease and low-grade acidosis (BiCARB): a pragmatic randomised, double-blind, placebo-controlled trial. BMC medicine. 2020;18(1):91.
